# Potential and Challenges for the Clinical Use of d-Serine As a Cognitive Enhancer

**DOI:** 10.3389/fpsyt.2018.00014

**Published:** 2018-02-05

**Authors:** Gerson D. Guercio, Rogerio Panizzutti

**Affiliations:** ^1^Instituto de Ciencias Biomedicas, Universidade Federal do Rio de Janeiro, Rio de Janeiro, Brazil; ^2^Global Brain Health Institute, Trinity College Dublin, Dublin, Ireland

**Keywords:** cognitive enhancer, biomarker, sodium benzoate, d-amino acid oxidase, glycine

## Abstract

After 25 years of its discovery in the rat brain, d-serine is a recognized modulator of synaptic plasticity and cognitive processes through its actions on the NMDA-glutamate receptor. Importantly, cognitive impairment is a core feature of conditions, such as schizophrenia, Alzheimer’s disease, depression, and aging, and is associated to disturbances in NMDA-glutamate receptors. The d-serine pathway has been associated with cognitive deficits and these conditions, and, for this reason, d-serine signaling is subject of intense research to probe its role in aiding diagnosis and therapy. Nevertheless, this has not resulted in new therapies being incorporated into clinical practice. Therefore, in this review we will address many questions that need to be solved by future studies, regarding d-serine pharmacokinetics, possible side effects, other strategies to modulate its levels, and combination with other therapies to increase its efficacy.

## Introduction

In a landmark study, Hashimoto and colleagues (1) discovered the presence of a substantial amount of d-serine in the rodent brain. In the following year, they reported that d-serine is present in high concentration in the human brain as well (2). Interestingly, it was later shown that d-serine is enriched in brain regions that contain a high concentration of the *N*-methyl-d-aspartate receptor (NMDAR), such as the cerebral cortex, hippocampus, amygdala, and retina (3).

The source of d-amino acids in mammals used to be attributed to diet or intestinal bacteria (4), until Wolosker et al. (5) identified serine racemase (SR) as the endogenous source of d-serine through racemization of l-serine. SR was first described to be exclusively present in astrocytes (5–8), but subsequent work has shown that SR is also present in neurons. Kartvelishvily et al. (9) demonstrated robust SR staining in neurons of the rat forebrain, and synthesis of d-serine by primary neuronal cultures. Additionally, a study using *in situ* hybridization confirmed that SR mRNA is predominantly expressed in rat brain neurons (10). Confirming a predominant neuronal expression, another group found the presence of SR in glutamatergic and GABAergic neurons of the mouse forebrain, but not in astrocytes (11).

In a more recent study, Benneyworth and colleagues (12) observed a 60% reduction in SR expression when SR was knocked out specifically in glutamatergic neurons. On the other hand, the knockout in the astrocytes caused a ~10% decrease in SR expression, while the remaining SR (~30%) was ascribed to other types of neurons. Importantly, *in vivo* work with microdialysis showed that neurons release d-serine (13). Finally, d-serine and SR are localized to neurons but not astrocytes in mouse and human brains (14). d-serine degradation is achieved through d-amino acid oxidase (DAAO), a flavin-dependent oxidase, resulting in the production of hydrogen peroxide, hydroxypyruvate, and ammonia (15). DAAO is especially enriched in the hindbrain, but it can also be found in the cortex and hippocampus, and it is present in glial cells and neurons (15).

The overlap between d-serine and NMDAR localization in the brain spurred investigations into a possible functional relationship between them. The NMDAR act as a coincidence-detector, as it requires not only binding of agonists but also depolarization of the postsynaptic membrane, which suspends the receptor blockade by Mg^2+^. The NMDAR is a tetrameric ion channel that may be composed by many configurations of three subunits, GluN1, GluN2, and less commonly, GluN3 (16). To be activated, the NMDAR requires simultaneous binding of the agonist glutamate to the GluN2 subunit and co-agonist glycine to GluN1. This binding is crucial for NMDAR activation, but later findings showed that d-serine is more potent both at binding to the co-agonist site and stimulating the receptor (17). Moreover, depletion of d-serine diminishes NMDAR activity (18) and long-term potentiation (LTP), a form of synaptic plasticity associated to learning and memory (19), and the relevance of d-serine to synaptic plasticity has been demonstrated in different brain regions (7, 20, 21).

Given the contribution of d-serine to LTP, and the fact that LTP is considered a key mechanism underlying learning and memory (22), it was no surprise when studies confirmed the importance of d-serine to learning and memory processes. For example, genetic inactivation of SR (23) and an acute stress protocol that diminishes d-serine levels (24) results in cognitive deficits. Importantly, the finding that the glycine modulatory site was not saturated *in vivo* (25) prompted investigations on whether exogenous d-serine administration could act as a cognitive enhancer. Remarkably, d-serine given intraperitoneally to rats increases NMDAR activation in the hippocampus (26), improves social memory in rats (27) and recognition and working memory in mice (28). Several animal studies now have confirmed the potential of d-serine as a cognitive enhancer, as well as its therapeutic potential in preclinical models. Here, we will review the evidence for the usefulness of d-serine cognitive-enhancing properties in different brain disorders and in age-related cognitive decline, including potential side effects and strategies to increase its efficacy.

## Schizophrenia

Schizophrenia is a severe neuropsychiatric disorder characterized by positive symptoms (hallucinations and delusions) and negative symptoms (apathy and avolition). Less known is the fact that most patients with schizophrenia also present cognitive impairments (29). Importantly, the degree of cognitive impairment is the best predictor of the daily functioning of a patient (30–32). Interestingly, many neurotransmitter systems important for cognition were found to be altered in schizophrenia, such as the dopaminergic, glutamatergic, cholinergic, and serotoninergic. These neurotransmitter systems are the target of most of the compounds evaluated for cognitive enhancement in schizophrenia, though none has been approved for clinical use.

Accumulating evidence indicates that the glutamate NMDAR might be hypoactive in schizophrenia. Pioneering studies with healthy human volunteers showed that infusion of different types of NMDAR antagonists induces a schizophrenia-like phenotype (33, 34). In addition, the NMDAR antagonist phencyclidine causes positive symptoms and deteriorates cognition in medication-free patients with schizophrenia (35). Interestingly, Steiner et al. (36) found a higher prevalence of NMDAR antibodies in the serum of acutely ill patients with schizophrenia, and polymorphisms in NMDAR subunits have been associated with the disorder (37). Finally, a recent study showed reduced protein levels of NMDAR subunits in *postmortem* samples of the dorsolateral prefrontal cortex in schizophrenia (38).

As discussed before, d-serine is the most potent endogenous co-agonist of the NMDAR. Remarkably, genetic mice models that present diminished d-serine levels recapitulate many aspects of schizophrenia, including sensorimotor gating and memory deficits (23, 39), reduced expression of BDNF (40), and brain ventricular enlargement (41). Notably, lower d-serine levels were found in blood, cerebrospinal fluid (CSF), and *postmortem* brain tissue of patients with schizophrenia (42–45). The decrease in d-serine levels in schizophrenia has been associated to increased levels of G72, a putative activator of DAAO (46–49). Accordingly, increased activity of DAAO has been found in postmortem samples of the cerebral cortex and the cerebellum in patients with schizophrenia (50, 51). Although negative findings have also been reported (52), a recent meta-analysis concluded that d-serine levels are reduced in the blood of patients with schizophrenia (53).

Considering that d-serine may be diminished in schizophrenia and its role in many brain processes affected in the disorder, several studies evaluated its efficacy as an add-on therapy to antipsychotic medication. Although generally safe, there are concerns about potential nephrotoxicity with d-serine (see Side Effects). Despite that, doses tested so far seem to converge to 30 and 60 mg/kg for historical reasons, as the first d-serine placebo-controlled trial for schizophrenia observed improvements in positive, negative, and cognitive symptoms with 30 mg/kg (54). However, while this dose has produced inconsistent results, 60 mg/kg or higher doses repeatedly resulted in therapeutic improvement, be it in chronic (55, 56) or prodromal patients (57). In fact, a recent meta-analysis showed that d-serine improved the positive and negative symptoms when added to antipsychotic drugs (53).

Another potential strategy to increase d-serine availability in the brain is reducing its degradation by DAAO. However, since DAAO is predominantly expressed in the midbrain, medulla, pons, and cerebellum and has relative low affinity for its substrates, some authors argue against a physiological role of DAAO in controlling d-serine availability in areas of the brain relevant for cognition and symptoms of schizophrenia (58). Yet several lines of evidence indicate that DAAO plays a role in controlling d-serine availability in the forebrain. Systemic administration of a DAAO inhibitor increased levels of d-serine in the rat cerebral cortex (59) and two different studies found increased levels of d-serine in the cerebral cortex and hippocampus of DAAO knockout mice (60, 61), although others studies did not replicate these findings (62, 63). Further evidence on a physiological role for DAAO in modulating cognition is provided by enhanced learning abilities of DAAO knockout mice (64, 65). Finally, in one clinical trial, the DAAO inhibitor sodium benzoate improved several symptoms and cognition in patients with chronic schizophrenia (66).

Other studies focused on glycine, the other endogenous NMDAR co-agonist. Initial studies with small samples found that very high doses of glycine (250 mg/kg or higher) reduced behavioral symptom severity in patients with schizophrenia (67, 68). Clinical use of glycine requires high doses because it does not readily cross the blood–brain barrier, which stimulated the study of drugs that could enhance extracellular glycine levels by inhibiting its reuptake. Accordingly, early studies with a small sample found that chronic treatment with sarcosine, an inhibitor of the glycine uptake transporter 1 (GlyT1), led to generalized improvements in symptoms of patients with schizophrenia (69, 70). Bitopertin is the first specific GlyT1 inhibitor, which showed potential in a Phase II trial (71), but a subsequent trial showed no significant improvements in primary outcomes (72). When interpreting the lack of effectiveness of the inhibition of GlyT in schizophrenia, it is important to highlight that electrophysiological data indicated that glycine, as opposed to d-serine, acts primarily at extra-synaptic NMDAR receptors, which are not required for LTP, and this might reduce the procognitive effect of enhancing glycine levels (73). We, therefore, believe that enhancement of d-serine levels poses a more suitable approach for development of new treatments for schizophrenia. In fact, a recent study compared the effect of chronic d-serine or bitopertin on mismatch negativity—an event-related potential to an odd stimulus in a sequence of similar stimuli—and on clinical symptoms. d-serine led to improvements on mismatch negativity, which correlated with changes in clinical symptoms. Bitopertin, on the other hand, did not change any of those measures (74).

An important question at this point is whether the improvements seen so far with the addition of d-serine will have real-life effects. This has not been generally investigated, but it would be a crucial finding to make the case for the use of d-serine in clinical practice. In contrast, increasing d-serine levels through DAAO inhibition with sodium benzoate was shown to improve quality of life and Clinical Global Impression (66). Interestingly, a recent study found that a combination of sodium benzoate and the GlyT1 inhibitor sarcosine improves cognition and global functioning of patients with schizophrenia, whereas sarcosine alone had no effect (75). However, because of the lack of a group of patients receiving sodium benzoate alone, we do not know whether there was a synergistic effect between the two compounds or the effects came from sodium benzoate only.

It is also important to consider which factors may or may not pair well with d-serine. For instance, there is evidence that d-serine is not effective when combined with clozapine compared to other antipsychotics (76), possibly because the mechanism of action of clozapine might include an increase in d-serine release (77). Indeed, clozapine treatment in patients with schizophrenia can increase plasma d-serine levels relative to l-serine (78). Conversely, it is reasonable to hypothesize that d-serine may lead to better outcomes when used in the subgroup of patients that have evidence of decreased d-serine signaling, a personalized approach not used so far.

d-Serine may also be useful in enhancing the effectiveness of other strategies to improve cognition in schizophrenia, such as cognitive or vocational training. To our knowledge, this has been tried only once, but the authors did not find any advantage of using d-serine along with 40 h of computerized cognitive training, as compared to training only (79). However, it is noteworthy that placebo produced pronounced effects, which may have obscured treatment-specific improvements, and the dose of 30 mg/kg d-serine used in the study has been previously shown to be ineffective to improve cognition in schizophrenia (55). Finally, the pharmacokinetics might play an important role, as d-serine has a short half-life of about 4 h (24, 55), and one can expect important fluctuations on blood levels after a single dose per day. Perhaps, it would be more advantageous to have an increase in d-serine concomitantly with the cognitive training. Animal studies could investigate this question specifically and provide valuable insight on how to increase d-serine effectiveness. An analogous approach has been tried with d-cycloserine, a partial NMDAR agonist. One study found that combined administration of d-cycloserine (once in a week) and a cognitive training (auditory discrimination training) led to better performance in the practiced training but failed to transfer its benefits to other untrained cognitive tasks (80). As the authors discuss, d-cycloserine has the disadvantage of being prone to cause tolerance, which may hinder its therapeutic effect in chronic treatments. However, this study is important because it shows that enhanced performance during training is not sufficient to enhance transfer of benefit to untrained cognitive tasks.

Furthermore, it is important to bear in mind that patients with schizophrenia generally live in an environment lacking sufficient cognitive stimulation, as they are typically unemployed and not pursuing education, partly because of untreated cognitive deficits. Merely stimulating the d-serine pathway to enhance neuroplasticity may be not enough to change maladaptive neural circuits formed throughout a patient’s life. For this reason, we believe that in the case of schizophrenia, therapies aimed at increasing d-serine signaling might prove more useful when combined with therapies that expose patients to learning experiences, such as cognitive training, which may induce the formation of more adaptive neural circuits.

## Age-Related Cognitive Decline

There has been a dramatic increase in the life expectancy of the world population in the last decades. Consequently, the rise of number of older adults is a global phenomenon that is becoming a challenge for public health. Aging is an important risk factor for many diseases, but even otherwise “healthy” older adults may present age-related cognitive decline (81). Aging is associated with declines in a number of cognitive domains, such as processing speed (82), memory (83), learning (84), working memory (85), executive function (86, 87). Importantly, declines have been found also in the primary processing of sensory input, such as visual processing (88), Gestalt detection (89), and speech processing (90). It is possible that declines in lower order processing of information (bottom-up) might contribute to declines in higher order processes (top-down), as degraded inputs may hamper the functioning of higher order circuits.

The age-related cognitive decline becomes important in older adults since it is associated with poorer quality of life, less independence (91), and higher incidence of falls (92, 93). Mobility is a crucial aspect of quality of life in older adults, and the cognitive decline can hamper the ability to drive, affecting social activities and independence, further contributing to depressive symptoms (94). As walking in our fast-paced and complex world requires attention, it is no surprise that cognitive deficits in older adults are associated to gait stability and falls (95). The association between cognition and different aspects of life makes it imperative to understand the underpinnings of the age-related cognitive decline and to develop new strategies for prevention.

In an effort to find molecular underpinnings associated to the age-related cognitive decline, studies in rodents revealed that aging is associated with reductions on the magnitude of LTP in the hippocampus, possibly because of alterations of NMDAR signaling (96). Several studies have revealed an age-related decline in the activation of NMDAR associated with a decrease in d-serine levels in the hippocampus (97, 98), possibly due to a decrease in SR expression (99). It is noteworthy that older LOU/C/Jall rats, which are resistant to age-related memory deficits (100), do not present a decrease in d-serine levels or SR expression with age (99). Finally, our group observed a negative association between plasma d-serine levels and age in healthy subjects (45). Putting together, these studies indicate that an age-related decrease in d-serine could contribute to the progression of the cognitive decline.

These findings raise the appealing possibility that increasing NMDAR activity might be of therapeutic value for the age-related cognitive decline. Accordingly, d-serine administration has been shown to improve cognition in older rodents and to correct many, though not all, of age-related declines in synaptic plasticity (101). From a clinical perspective, it is important to highlight that in a recent double-blind placebo-controlled crossover study our group observed that an acute oral administration of 30 mg/kg of d-serine improved spatial learning and problem solving, but not working memory, visual attention or cognitive flexibility, in older adults (102). Future studies should investigate whether higher doses of d-serine have a higher efficacy, and, crucially, whether a chronic treatment is tolerable and results in real-life effects, such as improved quality of life and reduced number of falls.

## Alzheimer’s Disease (AD)

Alzheimer’s disease is a chronic and progressive neurodegenerative disease that affects more than 6% of adults over 65 years of age worldwide (103), with an estimated global economic cost of $818 billion in 2015 (104). The pathophysiology involves synaptotoxicity, accumulation of extracellular β-amyloid (Aβ) aggregates and intracellular neurofibrils, gliosis, loss of neurons, and brain atrophy (105). Synaptic loss is critically involved in AD pathophysiology, and evidence indicates a possible causal role for glutamatergic dysfunction.

Activation of NMDAR may have different effects depending on the cellular location of the receptor. While LTP depends on activation of synaptic NMDAR, excessive activation of the extra-synaptic or synaptic NMDAR leads to high intracellular Ca^2+^ levels, which may cause cell death, a phenomenon termed excitotoxicity (73). For this reason, tight regulation of extracellular levels of glutamate is crucial. Astrocytes uptake glutamate from the extracellular space through different types of sodium-dependent excitatory amino acid transporters, and then glutamate is converted into glutamine by glutamine synthetase, transported back into the glutamatergic neuron, where it is hydrolyzed into glutamate by phosphate-activated glutaminase (106).

Evidence indicates that excessive NMDAR activation may contribute to AD pathology. Our group and others have shown that different forms of Aβ aggregates increase glutamate release from neurons and astrocytes, which leads to synaptic loss *via* inhibition of synaptic NMDAR currents and stimulation of extra-synaptic NMDAR currents (107–109). As reviewed in Rudy et al. (110), there are a plethora of studies linking AD pathology and an excess of glutamatergic activity, and, in line with this, memantine is a noncompetitive NMDAR antagonist approved for the clinical treatment of moderate to advanced AD.

As a result, dysfunctional d-serine metabolism could be associated to the increased NMDAR activity in AD and perhaps be a target for drug development. In fact, one study found that SR^−/−^ mice, which showed marked decrease in d-serine levels, are protected from injection of Aβ peptide, suggesting that d-serine could be a downstream element of Aβ toxicity (111). On top of that, it was shown that Aβ aggregates induce d-serine release, and d-serine levels are increased in animal models of AD (107, 112, 113). It could be the case that excess d-serine contributes to neuronal death in AD through excitotoxicity.

The question whether d-serine levels are altered in the brain in AD has been controversial. Studies in postmortem tissue found unaltered d-serine levels in different brain regions in AD, including the frontal, temporal, and parietal cortices (114–116). On the other hand, three different studies observed an increase in d-serine levels in the CSF of patients with AD, but the size of the differences between AD and controls varied greatly between studies (113, 117, 118).

It is tempting to speculate that the d-serine increase observed in the CSF of AD patients might be part of a protective mechanism to counter Aβ signaling and prevent AD pathology. Importantly, d-serine has been shown to increase neurogenesis and survival of newborn neurons (119) and to regulate apoptosis in a biphasic way, being able to inhibit it during its early-phases or stimulate it on later phases (120). This implies that increasing d-serine levels in the early-phases of AD might be therapeutically useful [while the NMDAR antagonist memantine is not effective in this early-phase of AD (121)]. The litmus test, then, is a clinical trial with patients with AD. Strikingly, a randomized, double-blind, placebo-controlled trial showed that 6 weeks of daily treatment with the DAAO inhibitor sodium benzoate improved cognitive composite and Clinician Interview Based Impression of Change plus Caregiver Input scores in patients in early-phase of AD (114). On the other hand, the clinical benefit of DAAO inhibition in AD may be mediated by an antioxidant effect, since d-serine degradation by DAAO generates hydrogen peroxide, one of the reactive oxygen species. Interestingly, there is evidence of increased DAAO levels in the peripheral blood of patients with mild cognitive impairment or AD, and the peripheral DAAO levels are positively associated with the severity of cognitive impairment (115). Moreover, in an animal model of AD, sodium benzoate attenuated oxidative stress and protected memory and learning (116). It is important to note, though, that the therapeutic effect of sodium benzoate might arise not only from its antioxidant effects but also from its immunomodulatory effects (122). In any case, if those clinical findings are replicated, sodium benzoate might prove to be a breakthrough for the treatment of patients in early-phases of AD.

## Depression and Anxiety

Major depressive disorder (MDD) is a multidimensional disorder characterized by at least one discrete depressive episode lasting at least 2 weeks and involving, among others, sleep disturbances, anhedonia, anxiety, feelings of worthlessness, and diminished ability to think and concentrate. In the US, MDD has a lifetime morbid risk of 29% (123), and its estimated annual cost is higher than US$ 80 billion (124). Notably, MDD is the second leading contributor to global disease burden, expressed in disability-adjusted years (125). Although cognitive impairment is a formal criterion item of a major depressive episode, its contribution to psychological suffering and functional outcome has been largely underappreciated. It is significant that the cognitive impairment persists after the resolution of an acute episode (126), and it is a predictor of functional outcome (127).

Animal models spurred the idea of an involvement of the NMDAR in the etiology of MDD, which gained momentum after the discovery that a single sub-anesthetic dose of ketamine elicits rapid and long-term antidepressant effects (128). Accordingly, preclinical and clinical work supports the idea of an overactivation of NMDAR in MDD (129), and different NMDAR antagonists show promise as potential antidepressants (130). However, a recent meta-analysis concluded that in adults with MDD ketamine has limited efficacy after 1 week of treatment, and the effects were even less pronounced after 2 weeks (131). Evidence was limited by risk for bias and the small number of participants and there were very limited data on issues like safety, tolerability, efficacy for cognition, quality of life, and costs to health-care services.

It is surprising that, in the same meta-analysis, the only other glutamate receptor modulators to show some efficacy in MDD was sarcosine, a glycine transporter inhibitor, that works by enhancing NMDAR activity (the opposite of ketamine) (131). Not only sarcosine, but also d-serine has shown antidepressant properties in both mice and humans (130, 132). In mice, acute d-serine administration has antidepressant and anxiolytic effect similar to ketamine (133), and chronic high levels of d-serine (through exogenous administration or overexpression of SR) reduced the proneness toward depression-related behavior (134). Accordingly, an acute single dose of d-serine improved mood in healthy human adults (135) and showed antidepressant-like effect in rats mediated by activation of AMPA-glutamate receptors and increased brain-derived neurotrophic factor, similar to that of ketamine (136). In addition, d-serine chronic administration can increase adult neurogenesis and survival of newborn neurons in mice (119) and regulate the functional synaptic integration of adult-born neurons (137), both processes that are associated to the therapeutic effect of antidepressants (138).

Consequently, despite our current incomplete understanding of the role of the NMDAR in MDD, data from rodents and humans warrants further research on the effect of d-serine administration in MDD patients. d-serine has a relatively safe profile, and its usefulness might be twofold, as it could improve both mood and cognition of the patients, hopefully giving them a better quality of life.

Interestingly, animal work has revealed that d-cycloserine can facilitate the extinction of fear memory, possibly because of the role of the NMDAR in synaptic plasticity and learning and memory (139). Building on this, many studies investigated whether d-cycloserine could facilitate the effectiveness of exposure-based therapy, which involves exposing the person to the feared context but in the absence of danger, so that relearning may occur (140). This effect was confirmed by a meta-analysis that showed that d-cycloserine can contribute to exposure-based therapy by increasing its efficiency, but the effects decrease over repeated sessions (141). More recently, d-cycloserine was shown to potentiate the effects of cognitive behavioral therapy in patients with anxiety disorders (142). Although results with d-cycloserine to promote the efficiency of behavior therapy are promising, this is a partial co-agonist of NMDAR with effects that diminish with the time. On the other hand, little is known about the effects of full co-agonists of NMDAR, such as d-serine or analogous agents, on the efficacy of behavior therapies in anxiety and depressive disorders.

## Side Effects

Although the majority of people do not experience side effects with d-serine, there is a concern that d-serine might induce nephrotoxicity in humans, as is the case with rats (143). Evidence indicates that nephrotoxicity is due to d-serine metabolism by DAAO, as rats that lack the enzyme do not develop glycosuria nor polyuria after high doses of d-serine (144). Therefore, co-administration of a DAAO inhibitor with d-serine may be a strategy to not only increase oral bioavailability of d-serine but also to prevent nephrotoxicity (145). This synergism has been observed mice, as treatment with a DAAO inhibitor rendered a small dose of d-serine (30 mg/kg) effective to treat prepulse inhibition deficits caused by the NMDAR antagonist dizocilpine, as opposed to the same dose of d-serine alone (146). It is conceivable that a combination of d-serine and sodium benzoate in future clinical trials will allow the use of lower doses of both drugs while retaining a high efficacy.

Alternatively, because d-serine and sodium benzoate have different pharmacokinetic and pharmacodynamic profiles, it is possible that each one of them might prove more effective and/or safe for different conditions. For instance, d-serine may be especially useful for depression because of its acute and chronic antidepressant effects, whereas sodium benzoate may be a safer approach in older adults with impaired renal function. In schizophrenia, a meta-analysis found that d-serine improves symptoms with small effect-sizes (*d* < 0.4), while one study found that higher doses of d-serine (60 mg/kg or higher) improve cognition with large effect-sizes (*d* > 1.0) (55). In contrast, in one study that warrants replication, twice daily administration of sodium benzoate (1 g/kg) improved cognition, symptoms and global functioning with large effect-sizes (all > 1.0) (66). Perhaps sodium benzoate had a higher efficacy because it not only inhibits DAAO but also modulates the immune system and has antioxidant properties, both of which may play a role in schizophrenia (147, 148). Future studies are needed to confirm the effectiveness of benzoate and its best doses for the treatment of schizophrenia.

## Conclusion and Perspectives

Pharmacological modulation of the d-serine pathway presents promising therapeutic opportunities for treatment of a variety of conditions that have in common cognitive and emotional disturbances. Specifically, d-serine and sodium benzoate are cheap and relatively safe drugs that have been administered to people taking a variety of other drugs. We believe future studies must aim to identify predictors of response across different conditions, in order to maximize the therapeutic effect of these drugs.

## Author Contributions

GG and RP designed, wrote, and reviewed the manuscript.

## Conflict of Interest Statement

The authors declare that the research was conducted in the absence of any commercial or financial relationships that could be construed as a potential conflict of interest.

## Abbreviations

AD, Alzheimer’s disease; CSF, cerebrospinal fluid; DAAO, d-amino acid oxidase; GlyT1, glycine uptake transporter 1; MDD, major depressive disorder; LTP, long-term potentiation; NMDAR, *N*-methyl-d-aspartate receptor; SR, serine racemase.
